# Identification of very early inflammatory markers in a porcine myocardial infarction model

**DOI:** 10.1186/s12917-019-1837-5

**Published:** 2019-03-12

**Authors:** Esther López, Francisco Miguel Sánchez-Margallo, Verónica Álvarez, Rebeca Blázquez, Federica Marinaro, Ana Abad, Helena Martín, Claudia Báez, Virginia Blanco, Verónica Crisóstomo, Javier García Casado

**Affiliations:** 1 Stem Cell Therapy Unit, Jesús Usón Minimally Invasive Surgery Centre, 10071 Cáceres, Spain; 2 CIBER de Enfermedades Cardiovasculares, 28029 Madrid, Spain

**Keywords:** Acute myocardial infarction, Porcine model, Early biomarkers

## Abstract

**Background:**

Acute myocardial infarction (AMI) is one of the most deleterious conditions leading to cardiovascular diseases and mortality. The importance of an early and accurate diagnosis assures immediate medical treatments, which are fundamental to reduce mortality and improve prognoses. AMI is associated to an inflammatory response which includes the increase of circulating inflammatory cytokines, chemokines and immune cell activation. This study aimed to identify which are the very early immune-related biomarkers that may be used as predictors of myocardial infarction severity. In order to mimic the pathophysiological events involved in human myocardial infarction, a temporary occlusion (90 min) of the mid-left anterior descending coronary artery was performed in a swine animal model.

**Results:**

Lymphocyte subsets analysis in peripheral blood revealed significant alterations in CD4+/CD8+ ratio and naïve and effector/memory T cell percentages at 1 h post-myocardial infarction. Changes in TH1/TH2-related cytokine, monocyte and neutrophil markers gene expression were observed in peripheral blood lymphocytes, as well. Additionally, significant correlations between cardiac parameters (cardiac enzymes, left ventricular ejection fraction and % infarct) and blood-derived parameters (cytokine expression and lymphocyte subset distribution) were found.

**Conclusions:**

Peripheral blood lymphocyte alterations are easily and swiftly detectable, so they may be good biomarkers for a very early prognosis and to predict myocardial infarction severity.

## Background

Acute myocardial infarction (AMI) is one of the main death causes in the world. Only in the United States, over 795,000 myocardial infarctions occur each year [1]. Consequently, an early and accurate diagnosis would guarantee immediate medical intervention, leading to reduced mortality and improved AMI prognosis.

Myocardial infarction results from the occlusion of a coronary artery and the subsequent myocardial ischemia. The generated ischemia results in cell death, initiating an inflammatory response ultimately resulting in scar formation [2]. During the inflammatory phase, chemokine and cytokine cascades activation results in leukocytes recruitment into the infarcted area. While neutrophils and macrophages are involved in removing dead cells and matrix debris from the wound site, activated macrophages release cytokines and growth factors, leading to granulation tissue formation [3]. These events, therefore, may be used as predictive biomarkers for early myocardial infarction detection.

Preclinical studies in cardiovascular research are necessary for the translation of basic research to the clinic. Animal models are widely used in the research of cardiovascular disease pathogenesis and drug therapy [4]. Swine are reliable animal models in the field of cardiovascular diseases, due to their similarity in cardiac function and anatomy with the human heart. As a matter of fact, cardiomyocyte metabolism, electrophysiological properties and response to an ischemic insult, such as AMI, have been reported to be closely similar to the human [5]. Among all the different surgical procedures developed to mimic acute or chronic myocardial infarction, minimally invasive approaches, like the closed-chest model, have been successfully developed using different coronary occlusion times [6]. The standardized endovascular model of 90 min balloon occlusion exhibits great similarities with AMI in humans [7].

Several evaluation methods and follow up procedures have been established for the swine myocardial infarction model. Biomarker tests combined with electrocardiographic analysis are the most used procedures for AMI diagnosis. Moreover, echocardiography and cardiac magnetic resonance imaging are commonly performed for the non-invasive detection of myocardial and scar mass, left ventricular volumes, geometric remodelling, scar reduction, contractility, myocardial perfusion and viable myocardial mass regeneration. On the other hand, cardiac enzymes can be detected in plasma at 4–6 h post-infarction and are the currently preferred diagnosis biomarkers for AMI [8]. The aim of this study was to identify the most significant alterations occurring during the very early phase of AMI in terms of cytokine expression and lymphocyte subset distribution in peripheral blood. So, tracing the first symptoms, together with detecting some biological biomarkers, could be useful to predict and monitor the pathogenic process of AMI. Here we propose some easily and rapidly accessible immunological markers that could provide early information for the diagnosis of AMI.

## Results

### Phenotypic analysis of peripheral blood lymphocytes

**Fig. 1:**
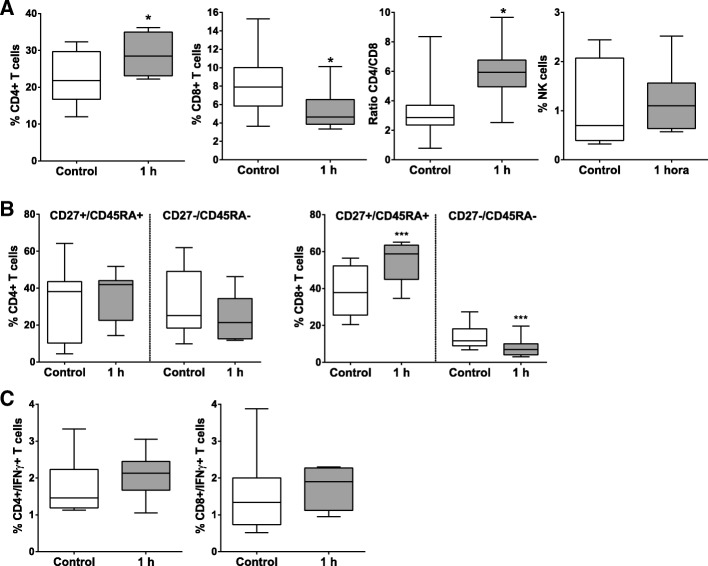
Lymphocyte subsets distribution in peripheral blood. Peripheral blood lymphocytes were isolated from blood samples collected before acute myocardial infarction model creation (control) and 1 h after it. The blood lymphocytes subsets (**a**). differentiation/activation T cell subsets (**b**) and IFNγ+ cells (**c**) were analyzed by flow cytometry. Paired comparisons were performed using a Student t-test for parametric data. Graphs show the mean ± SD (n = 11) of subpopulation subsets. * ≤ 0.05. ** ≤ 0.01. *** ≤ 0.001

The phenotypic analysis of peripheral blood lymphocytes (PBLs) in the animal model was analyzed by flow cytometry before and 1 h after myocardial infarction model creation. Statistically significant differences were found in peripheral blood T-cell subsets. The percentage of CD4+ T cells (gated as CD4+ CD8–) was significantly increased, while CD8+ T cells (gated CD4- CD8+) percentage decreased 1 h after myocardial infarction, in comparison to pre-AMI levels. Consequently, the CD4/CD8 ratio was significantly raised at 1 h after model creation. No statistically significant difference was found in NK cells (gated as CD3- CD8- CD4- CD16+) percentage (Fig. 1a).

A deep analysis of activation/differentiation markers was also performed on CD4+ and CD8+ T cell subsets. CD45RA and CD27 co-expression was analyzed on peripheral lymphocytes and the percentages of naïve T cells (CD45RA+ CD27+) and effector/memory T cells (CD45RA–CD27–) were compared before and 1 h after myocardial infarction. No significant changes were observed in CD4+ T cells. Nevertheless, the percentage of CD8+ naïve T cells and CD8+ effector/memory T cells exhibited substantial statistically significant differences (p‹0.001). While the CD8+ naïve T cell subsets significantly increased 1 h after myocardial infarction, the CD8+ effector/memory T cell subsets had a substantial decrease (Fig. 1b). Interferon γ (IFNγ) + T cells did not suffer significant changes 1 h after myocardial infarction, when compared to the control (Fig. 1c).

### Gene expression analysis of peripheral blood lymphocytes

**Fig. 2:**
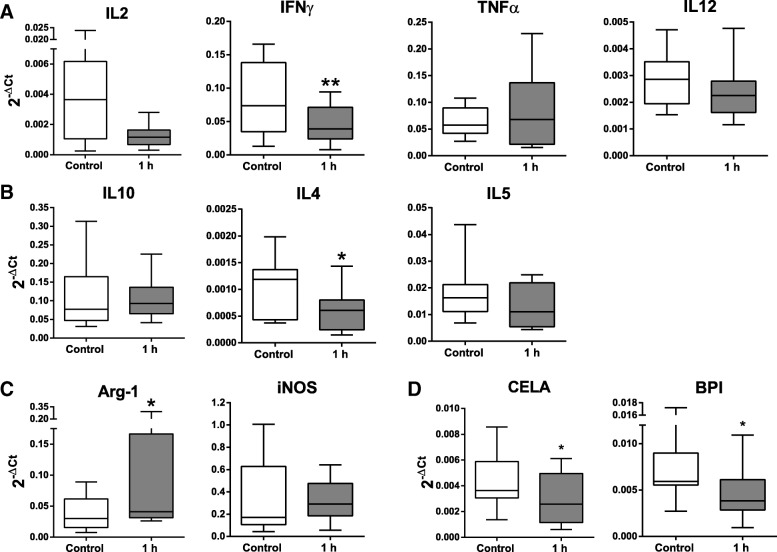
Cytokines gene expression in peripheral blood lymphocytes. TH1-related cytokines (**a**). TH2-related cytokines (**b**). monocyte markers (**c**) and neutrophil markers (**d**) were analyzed by qPCR. using GAPDH as a reference gene. Data were compared between control and 1 h after myocardial infarction. The statistical analysis was performed using a Student t-test for parametric data. Graphs show the mean ± SD (*n* = 11) of 2^-∆Ct^. * ≤ 0.05. ** ≤ 0.01

Cytokines and soluble factors gene expression of PBLs was evaluated by qPCR. The transcriptional analysis revealed that the most studied TH1-related cytokines (Interleukin (IL)2, IFNγ, Tumor Necrosis Factor α (TNFα), IL12) decreased 1 h after myocardial infarction, but only IFNγ showed significant differences (p‹0.01) (Fig. 2a). Regarding TH2-related cytokines, only IL4 gene expression changed significantly, showing a decrease 1 h after AMI (Fig. 2b).

Markers related with monocytes and neutrophils were also evaluated in this study. Among monocyte-related markers, a significant increase in Arg-1 was found (Fig. 2c). Surprisingly, gene expression of CELA and BPI (neutrophil markers), was significantly decreased at 1 h post-infarction (Fig. 2d).

### Cardiac function parameters and cardiac enzymes in myocardial infarction animal model

Myocardial infarction was successfully induced in all animals. A significant increase of cardiac enzymes (Creatine kinase-myocardial band (CK-MB) and Troponin I) was found at 24 h when compared to baseline levels. Cardiac Magnetic Resonance at day 7 after myocardial infarct induction showed a significant decrease of Left Ventricular Ejection Fraction (% LVEF) as well as a significant increase of myocardial infarction (Table 1).

**Table 1:** Data of cardiac function in terms of percentage of myocardial infarction and left ventricular ejection fraction and cardiac enzymes blood levels (Troponin I and CK-MB) in μg/l for the different animals

	Cardiac function		Troponin I (μg/l)			CK-MB (μg/l)		
Animal	% Infarct	% LVEF	Control	24 h	7 days	Control	24 h	7 days
#1	18	30	0	26	0.2	3.3	9.7	3
#2	24	38	0.018	18	0.8	2.2	7.9	3.5
#3	25	34	0.037	31	0.71	3.7	26	2.7
#4	28	22	0.071	22	0.29	5.5	15	4.4
#5	20	17	0.022	41	0.66	4.4	20	4.4
#6	25	26	0.017	18	0.13	4.3	19	3.4
#7	16	24	0.027	23	0.28	3	14	4.1
#8	23	18	0	17	0.039	2.8	6.6	2.3
#9	25	18	0	29	0.69	5.1	6.8	3.1
#10	26	14	0.024	52	0.71	2	6	2
#11	25	27	0	36	1.4	2.1	10	2
#12	20	19	0.013	44	0.78	2.6	21	4.5

### Correlation analysis between cardiac function and blood-derived parameters

Correlation analysis between cardiac parameters (cardiac enzymes, % LVEF, % Infarct) and immunological markers (T cell subsets and gene expression) was performed since it may be useful for the evaluation and follow-up of myocardial infarction in this animal model.

**Fig. 3:**
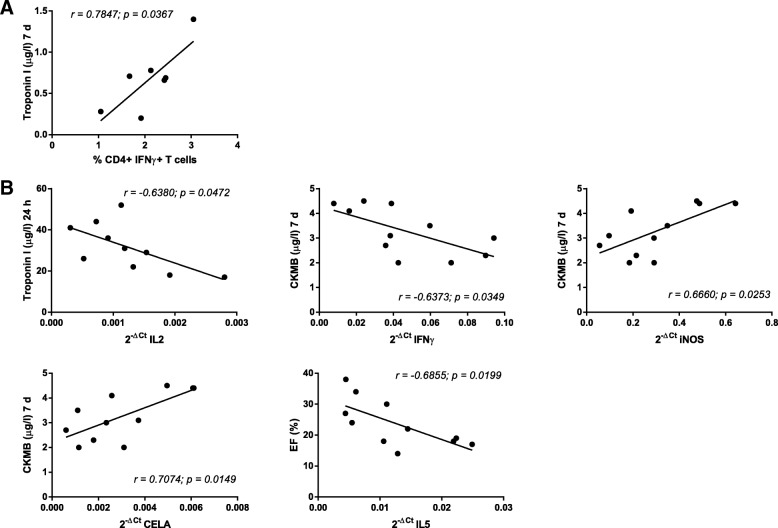
Correlation analysis between cardiac function parameters (cardiac enzymes. Left ventricular ejection fraction) and blood-derived parameters. Correlations were calculated using the Pearson coefficient correlation. Graphs show significant correlations observed between cardiac function parameters (blood levels of cardiac enzymes and percentage of left ventricular ejection fraction) and blood-derived parameters (lymphocytes subsets (**a**) and cytokine gene expression (**b**))

The analysis showed a strong positive correlation between the percentage of CD4+ IFNγ+ T cells 1 h after AMI and the levels of Troponin I at 7 days post-infarction (r = 0.7847; *p* = 0.0367) (Fig. 3a). More significant correlations were found between gene expression results and cardiac function parameters. In particular, CK-MB at 7 days and iNOS at 1 h (r = 0.6660; *p* = 0.0253) were found to be strongly and positively correlated, as well as CK-MB at 7 days and CELA at 1 h (r = 0.7074; *p* = 0.0149). Contrarily, strong negative correlations were identified between Troponin I at 24 h and IL2 at 1 h (r = − 0.6380; *p* = 0.0472), CK-MB at 7 days and IFNγ at 1 h (r = − 0.6373; *p* = 0.0349) and % LVEF at 7 d and IL5 at 1 h (r = − 0.6855; *p* = 0.0199) (Fig. 3b).

## Discussion

An earlier diagnosis of patients with symptoms suggestive of myocardial infarction might possibly reduce their morbidity and mortality. AMI is a life-threatening disorder and its rapid identification is important for the early initiation of appropriate, evidence-based and effective therapy. Diagnosis of AMI is usually based on electrocardiogram data and detection of biomarkers of myocardial injury, like cardiac enzymes. Although troponin assays have a good sensitivity, they have a low specificity due to troponin elevations in the absence of cardiac failure [9]. Consequently, it is necessary to identify early, effective and specific biomarkers for a rapid diagnosis of AMI.

Large animal models, such as swine, are widely accepted for studies of AMI and ischemic cardiomyopathy. They allow the identification of biomarkers under controlled conditions, avoiding the intrinsic variability of clinical studies: atherosclerosis, coronary arterial inflammation, myocardial inflammation and other associated pathologies. The homogeneity and repeatability of using an animal model under controlled conditions could also be considered a limitation because AMI in human medicine is frequently associated with risk factors and comorbidities (i.e. smoking, hypertension, diabetes or obesity).

AMI is defined as myocardial necrosis after a marked ischemia. Tissue injury generates endogenous signals that activate the innate immune system. The immune cells identify these signals and induce molecular pathways that lead to the recruitment of inflammatory cells in the healing infarct [10]. Our results have demonstrated that, 1 h after myocardial infarction, peripheral blood lymphocytes can “identify” these alarms showing alterations in lymphocyte subsets and cytokine expression.

More precisely, we have found that the ratio CD4+/CD8+ T cells increases due to an increase of percentage of CD4+ T cells and a decrease of CD8+ T cells. These findings are consistent with the results found in AMI patients diagnosed no more than 12 h from the onset of symptoms [11]. In this study, an analogous T cells-related gene expression was observed; however, the number of NK cells was reported as significantly decreased, while, in our animal model, NK cell subpopulations seemed not to be affected in the early stage of AMI.

Regarding lymphocyte differentiation/activation status, an increase in naïve T cells (CD8+ CD45+ CD27+) and a decrease in the effector/memory T cells (CD8+ CD45- CD27-) could be detected at 1 h post-infarction. The loss in peripheral blood CD8+ memory T-cell subsets may be caused by entrapment in the coronary microcirculation early after the onset of reperfusion, as shown in myocardial infarction patients [12]. Moreover, Emoto and cols. Related the regulatory T cells/effector T cells ratio with the pathophysiology of coronary atherosclerosis, suggesting that this ratio could be a useful marker for the evaluation of severity of atherosclerosis [13].

Cytokines are secreted as cellular signalling proteins that can mediate effector and regulatory effects on the immune response playing an essential role during T cell differentiation. Cytokine levels have been used as biomarkers for the diagnosis of multiple diseases such as rheumatoid arthritis [14], multiple sclerosis [15] or liver toxicity [16]. In this study, we have demonstrated that the gene expression of cytokines produced by Th1 lymphocytes (IFNγ) and Th2 lymphocytes (IL4) was altered at 1 h post-infarction. Clinical studies have demonstrated that both cytokines have a diagnostic value of success of percutaneous coronary intervention in patients with AMI [17].

Regarding peripheral blood monocytes, these cells migrate to the site of injure and infiltrate as macrophages. Using the coronary ligation technique in mice, it was observed that a large proportion of these newly recruited monocytes were provided by the spleen, increasing the blood monocytes before the recruitment into infarcted cardiac tissue [18]. This theory may explain, at least in part, the increased expression of Arg-1 in our animal model. Neutrophils are also used as predictive cells in coronary heart diseases [19] and their specific marker, myeloperoxidase (MPO), is reliable when used for discriminating AMI patients [20]. Our results have demonstrated a deficient expression of neutrophil marker BPI which, together with Arg-1, IFNγ and IL4, could contribute to the diagnosis and prognosis of AMI.

Additionally, in order to correlate lymphocyte subset distribution and gene expression levels with “classical” cardiac parameters, a correlation analysis was performed with the data from cardiac magnetic resonance imaging (% LVEF and Infarct area) and cardiac enzymes (Troponin I and CK-MB). In our analysis, serum Troponin I levels at 7 days post-infarction showed a positive correlation with CD4 + IFNγ+ lymphocytes, suggesting that patients with a high percentage of CD4 + IFNγ+ lymphocytes at 1 h post-infarction may have higher circulating Troponin I levels at 7 days. The correlation between IL2 and Troponin I levels indicates that a low expression level of IL2 at 1 h after infarction could be associated with a higher Troponin I level at 24 h. On the other hand, IFNγ, iNOS and CELA expression levels were also related with the serum levels of CK-MB at 7 days. Additionally, high expression levels of IL5 detected at 1 h post-infarction could be associated with a lower % LVEF at 7 days in patients.

## Conclusions

Although it is well known that cardiac enzymes are relevant and predictive biomarkers for the diagnosis of AMI, our swine myocardial infarction model -performed under controlled conditions- suggests that early inflammatory biomarkers may be useful in the follow-up of patients who have suffered an AMI. These biomarkers are easily and swiftly detectable, which means they could be candidates for early prognosis, predicting the severity of myocardial infarction in a clinical scenario. It is important to note that the usefulness of these biomarkers in AMI patients with concomitant immune-mediated diseases may be compromised, since immune biomarkers may be masked in patients with pre-existing immune disorders.

## Methods

### Animals and experimental design

Young female Large White pigs (*n* = 12) weighting 30–35 kg at the beginning of the study, were used for all experimental procedures. These animals were provided and housed by the Animal facility of the Jesús Usón Minimally Invasive Surgery Centre. At the end of experiments all animals were euthanatized by intravenous administration of 2 mmol/kg of potassium chloride. All experimental protocols were approved by the Ethics Committee on Animal Experiments of the Jesús Usón Minimally Invasive Surgery Centre, in accordance with the recommendations outlined by the local government (Junta de Extremadura) and the Directive 2010/63/EU of the European Parliament on the protection of animals used for scientific purposes.

### Myocardial infarction model creation

A closed chest reperfused myocardial infarction was created in the operating theaters of the Jesús Usón Minimally Invasive Surgery Centre as previously described [21]. Briefly, anesthetized animals were subjected to a percutaneous femoral access using a 7 Fr Introducer sheath (Terumo, Tokyo, Japan). A Hockey Stick 6 Fr guiding catheter (Mach 1, Boston Scientific Corporation, Natick, MA, USA) was navigated under fluoroscopic guidance to the origin of the left coronary artery. Coronary angiograms were obtained in the 40° left anterior oblique projection to better demonstrate the length of the left anterior descending artery. Subsequently, a coronary balloon catheter (typically 3 mm × 8 mm, Ryujin Plus, Terumo, Tokio, Japan) was inserted over a 0.014″ coronary guidewire and advanced to below the origin of the first diagonal branch, where it was inflated to occlude blood flow to the distal myocardium. Occlusion was maintained for 90 min to assure infarct creation. Upon balloon deflation, the coronary artery was checked for patency by repeating the angiogram. During the procedure, animals were fully monitored, including blood pressure, electrocardiogram, O_2_ saturation, and end tidal CO_2_ determinations. Continuous infusion of lidocaine at a rate of 1 mg/kg/h (Lidocaine, Braun Medical, Barcelona, Spain) was used throughout the procedure. Systemic heparin (Heparina Rovi 5%, Laboratorios farmacéuticos Rovi, Madrid, Spain) was intravenously injected (150 UI/kg) prior to percutaneous sheath placement.

### Phenotypic characterization of peripheral blood lymphocytes

Blood samples were drawn by puncture of the cranial vena cava before myocardial infarct induction and 1 h after the balloon deflation and collected in EDTA containing tubes for further analysis.

The PBLs were isolated by centrifugation over Histopaque-1077 (Sigma, St. Louis, MO). 1 × 10^6^ PBLs were stained with fluorescent-labeled monoclonal antibodies against extracellular porcine CD4, CD8α, CD14, CD16, CD27, CD45RA, CD56 and SLAII (AbD Serotec, Kidlington, United Kingdom). For the detection of intracellular antigen IFNγ, PBLs were fixed and permeabilized using Fix&Perm (Life technologies, Thermo Fisher Scientific Inc., Waltham, MA USA) and stained with the appropriate antibody (AbD Serotec). The cells were washed and re-suspended in PBS.

Flow cytometric analysis was performed in a FACScalibur cytometer (BD Biosciences, San Jose, CA, USA) after acquisition of 10^5^ events. Cells were primarily selected using forward and side scatter characteristics and fluorescence was analyzed using CellQuest software (BD Biosciences). Appropriate isotype-matched negative control antibodies were used in all the experiments.

### Gene expression analysis

For transcriptional analysis studies, total RNA from isolated lymphocytes was extracted and purified by using the mirVana miRNA Isolation Kit (Life technologies, Thermo Fisher Scientific Inc.), following the manufacturer’s instructions. RNA quality was evaluated through spectrophotometry and cDNA was synthesized from 1 μg of RNA in reverse transcription reaction with the iScript Reverse Transcription Supermix (BioRad, Hercules, California, USA). qPCR was performed with commercial gene expression assays (Life Technologies, Thermo Fisher Scientific Inc.) (Table 2), using TaqMan Fast Advance Master Mix in a QuantStudio 3 System (Applied Biosystems, Thermo Fisher Scientific). The qPCR products were quantified by fluorescent method using the 2^-∆Ct^ expression. Duplicates of all samples were analyzed separately and normalized using GAPDH as a reference gene.

**Table 2:** Gene and commercial references from gene expression assays

Gene	Life Techonologies Assay ID
IL-2	Ss03392428_m1
IFN-γ	Ss03391054_m1
TNF-α	Ss03391318_g1
IL-12A	Ss03391176_m1
IL-10	Ss03382372_u1
IL-4	Ss03394125_m1
IL-5	Ss03394369_m1
FOXP3	Ss03376695_u1
Nos2	Ss03374608_u1
Arg1	Ss03391394_m1
CELA1	Ss03392393_m1
BPI	Ss04321426_m1
GAPDH	Ss03375629_u1

### Cardiac magnetic resonance

Cardiac Magnetic Resonance studies (Intera 1.5 T, Philips Medical Systems. Best, The Netherlands) were performed at day 7 post-infarction. Retrospective cardiac triggering was used. A 4 elements phase array coil was placed around the animals’ chest. Images were acquired in the intrinsic cardiac planes: short axis, vertical long axis and horizontal long axis views. In order to measure left ventricular function and mass breath hold gradient, echo cine images were obtained over the entire left ventricle. For infarct size measurements, short axis images were acquired 5 to 15 min after the injection of 0.2 mmol/kg of a gadolinium-based contrast agent (Gadobutrol. Gadovist 1.1 mmol/l, Bayer Schering Pharma AG, Berlin, Germany) using a breath-hold 3D gradient-echo inversion-recovery sequence.

### Statistical analysis

Data were statistically analyzed with SPSS-21 software (SPSS, Chicago, IL, USA). Normality was assessed using a Shapiro-Wilk test. Paired comparisons were performed using a Student t-test for parametric data or Wilcoxon sign test for non-parametric data. Correlations were calculated using the Pearson coefficient correlation and “r” value was interpreted as follows: − 1.0 to − 0.5 or 0.5 to 1.0: Strong correlation, − 0.5 to − 0.3 or 0.3 to 0.5: Moderate correlation, − 0.3 to − 0.1 or 0.1 to 0.3: Weak correlation, − 0.1 to 0.1: None or very weak correlation. All *p*-values ≤0.05 were considered statistically significant.

## Abbreviations

AMI: Acute myocardial infarction
CK-MB: Creatine kinase-myocardial band
IFNγ: Interferon γ
IL: Interleukin
LVEF: Left ventricular ejection fraction
PBLs: Peripheral blood lymphocytes
TNFα: Tumor Necrosis Factor α
